# Incidence and risk factors of oral mucosal pressure injury in patients with oral tracheal intubation: systematic review and meta-analysis

**DOI:** 10.3389/fmed.2026.1783726

**Published:** 2026-04-13

**Authors:** Yating Gao, Li Zhang, Qingjie Zhu, Qiansheng Jin, Jiangquan Yu, Ruiqiang Zheng

**Affiliations:** 1 Department of Medical Nursing, Yangzhou University, Yangzhou, Jiangsu, China; 2 Department of Critical Care Medicine, Northern Jiangsu People’s Hospital, Yangzhou University, Yangzhou, Jiangsu, China

**Keywords:** endotracheal tube, incidence, meta-analysis, oral mucosa pressure injury, risk factors

## Abstract

**Background:**

To systematically review the incidence and risk factors of oral mucosal pressure injury in patients with oral tracheal intubation.

**Methods:**

Two researchers independently searched eight databases, PubMed, Embase, Web of Science, Cochrane Library, China National Knowledge Infrastructure (CNKI), WanFang Database, VIP Database and China BioMedical Literature Database (CBM) from inception to February 27th, 2026. Data analyses were performed using Stata 18.0 software.

**Result:**

Twenty-four studies (9,904 patients) were included in the meta-analysis. The incidence of oral mucosal pressure injury in patients undergoing oral tracheal intubation was 29%. Risk factors include higher APACHE II score[OR 1.32, 95%CI:1.19–1.50], diabetes [OR 5.31, 95%CI: 1.88–14.87], serum albumin [OR0.37,95%CI:0.2–0.68], use of anticoagulants [OR 1.68, 95%CI: 1.02–2.77], use of sedative drugs [OR 3.35, 95%CI: 2.16–5.21], use of vasoactive drugs [OR 1.55, 95%CI: 1.25–1.92], prone position ventilation [OR 3.94, 95%CI: 2.64–5.93], prolonged tracheal intubation indwelling time [OR 1.08, 95%CI: 1.05–1.12], use of hard dental pads [OR 3.22, 95%CI: 2.25–4.66], tracheal intubation model [OR 2.72, 95%CI: 1.62–4.62].

**Conclusion:**

The incidence of oral mucosal pressure injury is relatively high in patients with oral tracheal intubation, and there are many risk factors. Nursing staff should enhance their awareness of oral mucosal pressure injury in patients with oral tracheal intubation. They should accurately identify high-risk groups at an early stage based on risk factors and formulate targeted and personalized preventive measures to reduce the risk of injury.

**Systematic review registration:**

https://www.crd.york.ac.uk/PROSPERO/view/CRD420251062658, identifier PROSPERO (CRD420251062658).

## 1 Introduction

Oral mucosal pressure injury (OMPI) caused by oral tracheal intubation is the most common medical device-related pressure injury (1). It is mainly caused by the pressure and friction exerted on the oral mucosa by tracheal intubation and fixation devices, and often occurs on the mucous membranes of the hard palate, lips, cheeks, and tongue and abdomen in the oral cavity (2–4). Generally, the indwelling time of tracheal intubation in critically ill patients is relatively long, and the oral observation space is limited and the injury site is relatively concealed after intubation. Therefore, oral mucosal pressure injury (OMPI) caused by oral intubation is easily overlooked. OMPI is prone to cause adverse outcomes such as pain, infection, tissue adhesion and functional disorder in patients. This not only prolongs the hospital stay of patients and affects the prognosis of the disease, but also increases the workload of medical staff (5–7).

In recent years, there have been an increasing number of studies on OMPI for patients with oral tracheal intubation in the intensive care unit (2, 8, 9). Due to differences in research areas, assessment tools, oral care measures, sample sizes, etc., there are significant differences in current on the incidence of OMPI in patients with oral tracheal intubation. The incidence rate of OMPI ranges from 2.59 to 80.14% (10, 11). Existing studies have found that there are many potential influencing factors associated with the occurrence and development of OMPI in patients with oral tracheal intubation. However, the influencing factors reported by different studies vary. Therefore, this study systematically integrated the incidence and risk factors of OMPI in patients with oral tracheal intubation through a meta-analysis. Providing evidence-based evidence for the formulation of standardized prevention strategies in clinical practice and the reduction of the risk of OMPI occurrence.

## 2 Methods

### 2.1 Design and search methods

This study was prospectively registered on the International Prospective Register of Systematic Reviews platform (PROSPERO number: CRD420251062658) to ensure transparency and avoid reporting bias. By combining subject terms with free terms, domestic and foreign databases were systematically retrieved, including PubMed, Embase, Web of Science, Cochrane Library, China National Knowledge Infrastructure (CNKI), WanFang Database, VIP Database and China BioMedical Literature Database (CBM). The search terms are “Intubation, Intratracheal/Intratracheal Intubation/Intratracheal Intubations/Intubations, Intratracheal/Intubation, Endotracheal/Endotracheal Intubation/Endotracheal Intubations/Intubations, Endotracheal/Mechanical Ventilation/Artificial airway” “Pressure Ulcer/Pressure Ulcers/Ulcer, Pressure/medical device related pressure injuries/device-related ulcer/Decubitus Ulcer/Decubitus Ulcers/Ulcer, Decubitus/Pressure Sore/Pressure Sores/Sore, Pressure/Decubitus Sore/Decubitus Sores/Sore, Decubitus/Pressure Injury/Injury, Pressure/Pressure Injuries/oral mucosal pressure injury/mucosal pressure injury” “Risk Factors/risk factor/factor*, social risk/risk factor*, social/health correlates/population*at risk/risk score*/risk factor score*/influence*factor*/relevant factor*/predict*factor*”. The retrieval period is from the establishment of the database to February 27th, 2026.

### 2.2 Inclusion and exclusion criteria

Inclusion criteria: (1) The study type is cross-sectional study, case–control study, or cohort study; (2) Patients aged 18 years or older who underwent oral tracheal intubation and had no oral mucosal injury before intubation; (3) In the research content, the analysis data of the incidence and influencing factors of OMPI in patients with oral tracheal intubation can be obtained, including odds ratio (OR), mean plus or minus standard deviation ( $\bar{X}\pm S$ ), 95% confidence interval (95%CI), or the original data that can be converted to the above data can be provided.

Exclusion criteria: (1) Repeated publication or data from the same study; (2) Complete data or the full text cannot be obtained; (3) Reviews, conference papers and animal experiments; (4) Low quality of the article; (5) Animal experiments.

### 2.3 Literature screening

The retrieved literature was imported into the Note Express software to remove duplicate literature. Two researchers independently conducted literature screening and data extraction and cross-checking. If there were any differences, they discussed and decided with the third researcher. Researchers first read the title and abstract to eliminate obviously irrelevant literature, and then read the full text to determine the literature to be finally included.

### 2.4 Data extraction and quality assessment

The data extraction content includes the first author, publication year, survey time, country, research type, etc. Two researchers independently used the Newcastle-Ottawa Scale (NOS) to evaluate the literature quality of case–control studies and cohort studies. The content included three aspects: the selection of research subjects, comparability between groups, and measurement of results or exposure factors. A score of ≤3 was considered low-quality literature. Literature rated between 4 and 6 is of medium quality, while literature ≥7 is of high quality (12). The Quality of the cross-sectional study was evaluated using the bias risk assessment criteria of the Agency for Healthcare Research and Quality (AHRQ) in the United States: 0–3 was rated as low quality, 4–7 as medium quality, and 8–11 as high quality (13). If there are differences of opinion, it should be determined after discussion with the third researcher.

### 2.5 Data analysis

The data were analyzed by using Stata 18.0 software. Each variable was combined using the odds ratio (OR value) and its 95% confidence interval (95%CI) as the effect size. *p* < 0.05 was considered statistically significant. The size of heterogeneity was evaluated using *I*^2^and *Q* tests. When *I*^2^ ≤ 50% and *p* ≥ 0.1, a fixed-effect model was selected to combine the effect size, indicating that the heterogeneity was acceptable. Conversely, it indicates significant heterogeneity. Identify the sources of heterogeneity and combine the effect sizes using a random effects model. Publication bias was evaluated using a funnel plot combined with Egger’s test, with a test level of *α* = 0.05.

## 3 Results

### 3.1 Literature search results

**Figure 1:**
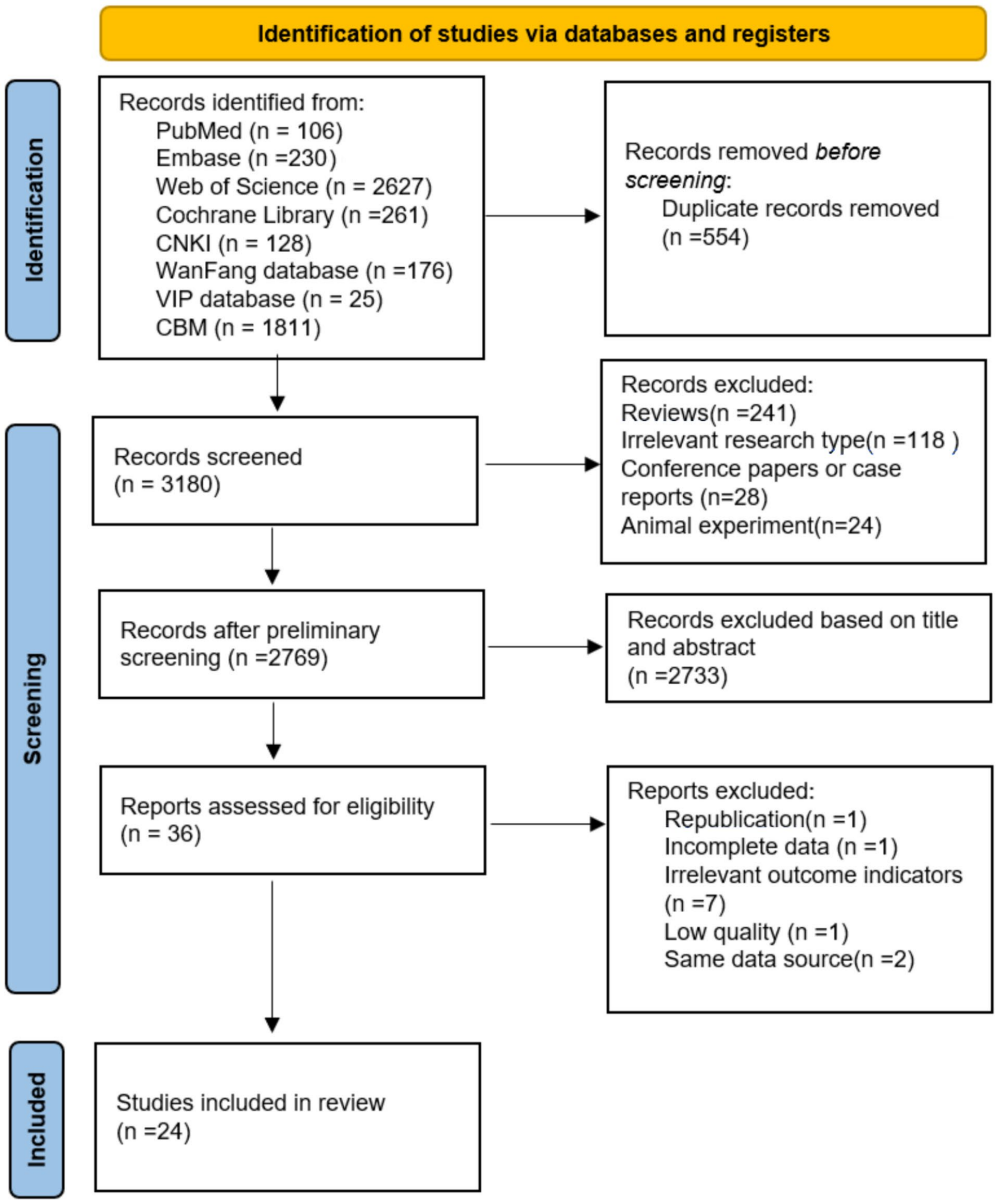
Flow chart of literature screening.

A total of 3,734 studies were retrieved from the eight databases. After eliminating 544 duplicate documents, 36 were screened out after reading the titles and abstracts of the documents. After reading the full text, 24 were included (2, 3, 5, 8, 10, 11, 14–31). The study selection flowchart is presented in Figure 1.

### 3.2 Characteristics of the included literature

The total sample size of 24 studies included was 9,904 cases, including 2,576 cases in the case group and 7,328 cases in the control group. Eleven articles (5, 15–17, 20, 21, 23, 24, 26, 29, 31) evaluated OMPI using the Reaper Oral Mucosa Pressure Injury Scale (ROMPIS) developed by Reaper et al. (32). Three paper (22, 27, 30) proposed the M-ROMPIS evaluation scale using Fitzgerald et al. (33). One paper (18) used the modified Oral Assessment Guide (OAG) by Barnason et al. (34). Two articles (3, 25) used self-developed assessment scales, while 7 articles (2, 8, 10, 11, 14, 19, 28) classification is carried out by nurses according to the National Pressure Ulcer Advisory Group (NPIAP) staging. The included 52 risk factors. The basic characteristics and quality evaluation results of the included literature are shown in Table 1.

**Table 1:** Characteristics and quality assessment of included studies (*n* = 24).

Author	Year	Survey period	Country	Study design	Research object	Sample size (Case group/Control group)	Evaluation frequency (day)	Influencing factors	NOS/AHRQ scores
Xia (27)	2026	2024.01–12	China	Cohort study	Emergency EICU	36/307	—	1, 2, 3, 4, 5, 6	5
Wang et al. (28)	2026	2022.12–2024.12	China	Case-control study	Emergency EICU	35/130	2	2, 4, 7, 8	5
Ai et al. (30)	2025	2023.04–12	China	Cohort study	ICU	551/968	4	9, 10, 11	8
Cai et al. (29)	2025	2024.05–12	China	Cohort study	ICU	235/343	4	3, 4, 12, 13, 14	7
Zhao et al. (31)	2025	2024.03–08	China	Cohort study	ICU	36/114	2	4, 8, 15	6
Ozdemir and Kavakli (2)	2025	2022.01–05	Turkey	Cohort study	ICU	104/146	3	16, 17	7
Yang (26)	2025	2024.05-10	China	Case-control study	Emergency EICU	18/65	—	3, 4, 6, 12, 18, 19, 20, 21	7
Zhang et al. (22)	2025	2021.01–2024.03	China	Case-control study	Emergency EICU	53/156	—	4, 6, 8, 22, 23	7
Chen et al. (8)	2025	2019.08–2020.08	China	Case-control study	ICU EICU	104/108	—	4, 10, 24, 25	6
Li et al. (15)	2025	2023.07–2024.07	China	Cohort study	ICU	181/239	4	14, 26, 27, 28	7
Hu et al. (14)	2025	2022.06–2024.03	China	Case-control study	ICU	103/135	—	4, 29, 30, 31	6
Zhang et al. (17)	2024	2022.01–2023.08	China	Cohort study	ICU Elderly patients	103/430	2	4, 6, 8, 22, 30, 32, 33	8
Wang et al. (16)	2024	2023.01–05	China	Cohort study	ICU	286/354	—	4, 8, 15, 22, 34, 35, 36	8
Wang et al.(20)	2024	2019.01–2023. 12	China	Cohort study	ICU Elderly patients	29/168	—	4, 18, 33, 37, 38	7
He et al. (23)	2024	2022.07–2023.03	China	Case-control study	Emergency EICU	33/69	—	2, 4, 6, 8, 18, 19, 39	7
Li et al. (24)	2024	2022.12–2023–10	China	Cohort study	ICU	99/453	2	3, 4, 8, 12, 33, 40, 41	7
Gu et al. (25)	2024	2023.01–2023.12	China	Cohort study	ICU	71/407	1	1, 3, 4, 12, 16, 42	8
Chen et al. (21)	2024	2023.01–06	China	Cohort study	ICU	135/320	—	44, 45, 46, 47, 48	6
Cambaz et al. (11)	2024	2021.03–2022.06	Turkey	Cohort study	ICU	117/29	1	49	8
Yu and Wu (19)	2023	2022.01–09	China	Cohort study	ICU	144/301	—	1, 3, 4, 6, 12, 33	6
Liu et al. (3)	2022	2021.01–11	China	Cohort study	EICU	31/85	2	4, 6, 8, 14, 15, 18, 22, 33, 50	6
Choi et al. (5)	2020	2017.09 2019.03-05	Korea	Cohort study	ICU	15/12	1	6, 12, 31, 33, 51, 52	8
Hampson et al. (10)	2018	2010.10–2013.06 2013–07–2016.03	Australia	Case-control study	ICU	52/1956	—	—	6
Wickberg and Falk (18)	2017	2014.02–2014.07	Sweden	Cohort study	EICU	5/33	1	4	7

“–” indicates not mentioned in the text. 1. History of diabetes; 2. Tracheal intubation model; 3. RASS score (Resting-Sedation score); 4. Indwelling time of tracheal intubation; 5. Braden Scale score; 6. Vasoactive drugs; 7. Oral secretions; 8. APACHEII score (Acute Physiology and Chronic Health II Score); 9. Nutritional therapy; 10. C-reactive protein; 11. Procalcitonin; 12. Dental pads; 13. BOAS score (Short Head Obstructive Airway Syndrome Score); 14. Platelets; 15. Sedative drugs; 16. Frequency of oral care; 17. Parenteral nutrition; 18. Anticoagulants; 19. Hypoproteinemia; 20. MEWS score (Modified Early Risk Score); 21. Lactic acid; 22. Prone position ventilation; 23. When intubating, one cannot file a chief complaint; 24. Cardiovascular diseases; 25. Death; 26. Sepsis; 27. Materials of dental pads; 28. Probiotics; 29. Braden Scale score; 30. Hemoglobin; 31. Hematocrit (hematocrit); 32. Gender; 33. Serum albumin; 34. Improve the Beck Oral score; 35. Oxygenation index; 36. Fixed frequency of intubation; 37. GCS score (Glasgow Coma Scale); 38. *Pseudomonas aeruginosa* in the oral cavity; 39. Anemia; 40. Age; 41. Senior doctor; 42. Tracheal intubation materials; 43. Frequency of oral aspiration; 44. Use ECMO after the operation; 45. History of hypertension; 46. The lowest intraoperative body temperature; 47. Preoperative hematocrit; 48. Preoperative fasting blood glucose; 49. BMI (Body Mass Index); 50. APTT; 51. Use corticosteroids; 52. Commercial intubation fixator.

### 3.3 The incidence of oral mucosal pressure injury in patients with oral tracheal intubation

**Figure 2:**
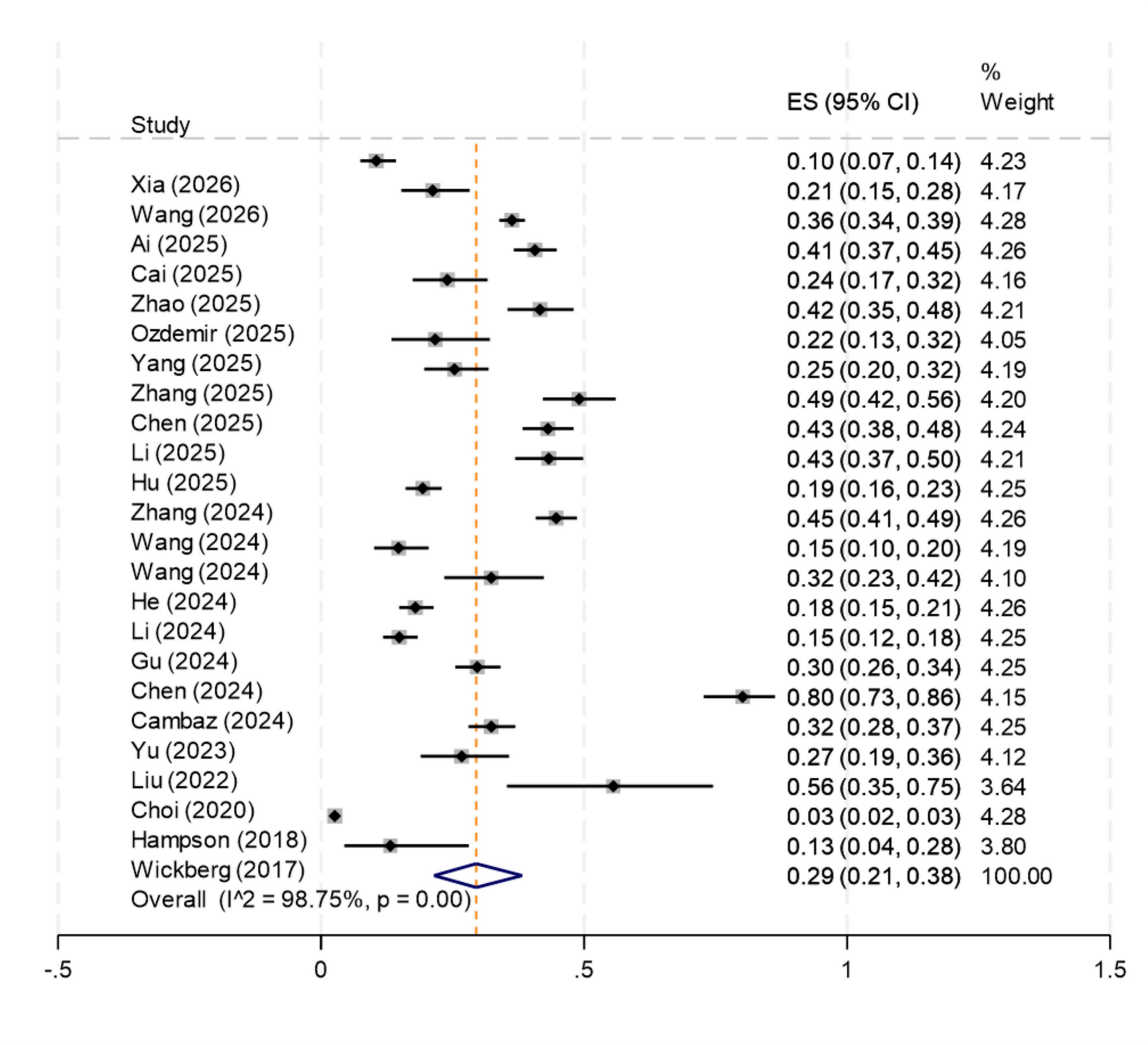
Meta-analysis forest plot of incidence of oral mucosal pressure injury in patients undergoing endotracheal intubation.

Due to the high heterogeneity of the 24 included literatures (*I*^2^ = 98.75%, *p* < 0.001), the random effects model was selected for analysis. The results showed that the incidence of OMPI in patients with oral tracheal intubation was 29% (95%CI: 21% ~ 38%), as shown in Figure 2. To investigate the sources of heterogeneity, we conducted subgroup analyses across seven dimensions. The dimensions analyzed included the fixed type, country, study design, OMPI assessment scale, assessment frequency, sample size, and research object. The specific results of the subgroup analysis are shown in Table 2.

**Table 2:** Meta-regression and subgroup analysis results on the incidence of oral mucosal pressure injury in patients undergoing endotracheal intubation.

Group	Subgroup type	Studies (n)	Heterogeneity test results		Effect model	The incidence rate of OMPI (%, 95%CI)	*P*
			*I^2^* (%)	*P*			
Fixed type	Not mentioned	5	98.56	<0.001	Random	32(10 ~ 60)	<0.001
	Traditional fixation	12	93.16	<0.001	Random	36(30 ~ 41)	<0.001
	Decompression fixation	7	98.18	<0.001	Random	18(8 ~ 30)	<0.001
Research countries	China	19	96.14	<0.001	Random	28(23 ~ 34)	<0.001
	Other countries	5	99.45	<0.001	Random	35(4 ~ 76)	<0.001
Research type	Cohort study	17	97.29	<0.001	Random	31(24 ~ 38)	<0.001
	Case–control	7	99.04	<0.001	Random	26(9 ~ 48)	<0.001
Evaluation Scale	ROMPIS	11	95.76	<0.001	Random	30(23 ~ 38)	<0.001
	NPIAP	7	99.47	<0.001	Random	37(14 ~ 63)	<0.001
	Others	6	97.08	<0.001	Random	21(11 ~ 32)	<0.001
Evaluation frequency	≤2 Twice/d	8	97.31	<0.001	Random	30(17 ~ 46)	<0.001
	>2 Twice/d	5	95.66	<0.001	Random	36(28 ~ 44)	<0.001
	Not mentioned	11	99.11	<0.001	Random	16(14 ~ 41)	<0.001
Sample size	<500	17	96.40	<0.001	Random	30(23 ~ 39)	<0.001
	>500	7	99.54	<0.001	Random	27(12 ~ 46)	<0.001

### 3.4 Risk factors for oral mucosal pressure injury in patients with oral tracheal intubation

A meta-analysis was conducted on the risk factors involving ≥2 literatures, and a total of 13 risk factors were included for analysis, as shown in Table 3.

**Table 3:** Meta-analysis results of risk factors for oral mucosal pressure injury in patients undergoing endotracheal intubation.

Factors	Studies (n)	Sample (n)	Heterogeneity test results		Effect model	Results of meta-analysis	Egger’s test		
			*I^2^* (%)	*P*		OR (95%CI)	*P*	*t*	*P*
APACHEII Score	8	2,431	84.9	<0.01	Random	1.32 (1.19–1.50)	<0.01	2.03	0.089
Indwelling time of tracheal intubation	15	4,019	93.3	<0.01	Random	1.08 (1.05 ~ 1.12)	<0.01	1.1	0.293
RASS Score	6	1,606	86.5	<0.01	Random	1.34 (0.65–1.95)	0.65	0.27	0.797
Serum albumin (g/L)	5	1,158	96.3	<0.01	Random	0.37 (0.20–0.68)	<0.01	−1.27	0.293
Hemoglobin (g/L)	2	539	92.2	<0.01	Random	0.94 (0.80–1.01)	0.09	—	
Vasoconstrictive drugs	5	1,110	0	0.521	Fixed	1.55 (1.25–1.92)	<0.01	0.93	0.422
Prone position ventilation	3	1,222	33.9	0.22	Fixed	3.94 (2.64–5.93)	<0.01	−0.53	0.692
Hypoproteinemia (<35 g/L)	2	185	0	0.696	Fixed	4.9 (1.95–12.30)	<0.01	—	
Diabetes	2	780	33.8	0.219	Fixed	5.31 (1.88–14.87)	<0.01	—	
Use hard dental pads	5	1,381	0	0.679	Fixed	3.22 (2.25–4.66)	<0.01	0.21	0.847
Use anticoagulant drugs	4	498	0	0.573	Fixed	1.68 (1.02–2.77)	0.03	−1.52	0.269
Use sedative drugs	3	906	0	0.835	Fixed	3.35 (2.16–5.21)	<0.01	0.19	0.879
Tracheal intubation model	2	169	37.1	0.207	Fixed	2.72 (1.62–4.62)	<0.01	—	

When the sample size of the bias test is less than 3, Egger’s results cannot be obtained, so “–” is used.

### 3.5 Sensitivity analysis and publication bias test

Sensitivity analysis was conducted by eliminating individual studies one by one. After combining the effect sizes, there was no significant change, indicating that the results of the incidence rate was relatively stable. Publication bias was determined by Egger’s test, and the results showed that there was no publication bias (*p* = 0.106 > 0.05). Sensitivity analysis was conducted on the risk factors with statistical significance (*p* < 0.05) in the meta-analysis results, and the results showed that they were relatively stable. Egger’s test results indicated that the results of this meta-analysis were relatively stable and there was no obvious publication bias, as shown in Table 3.

## 4 Discussion

**Figure 3:**
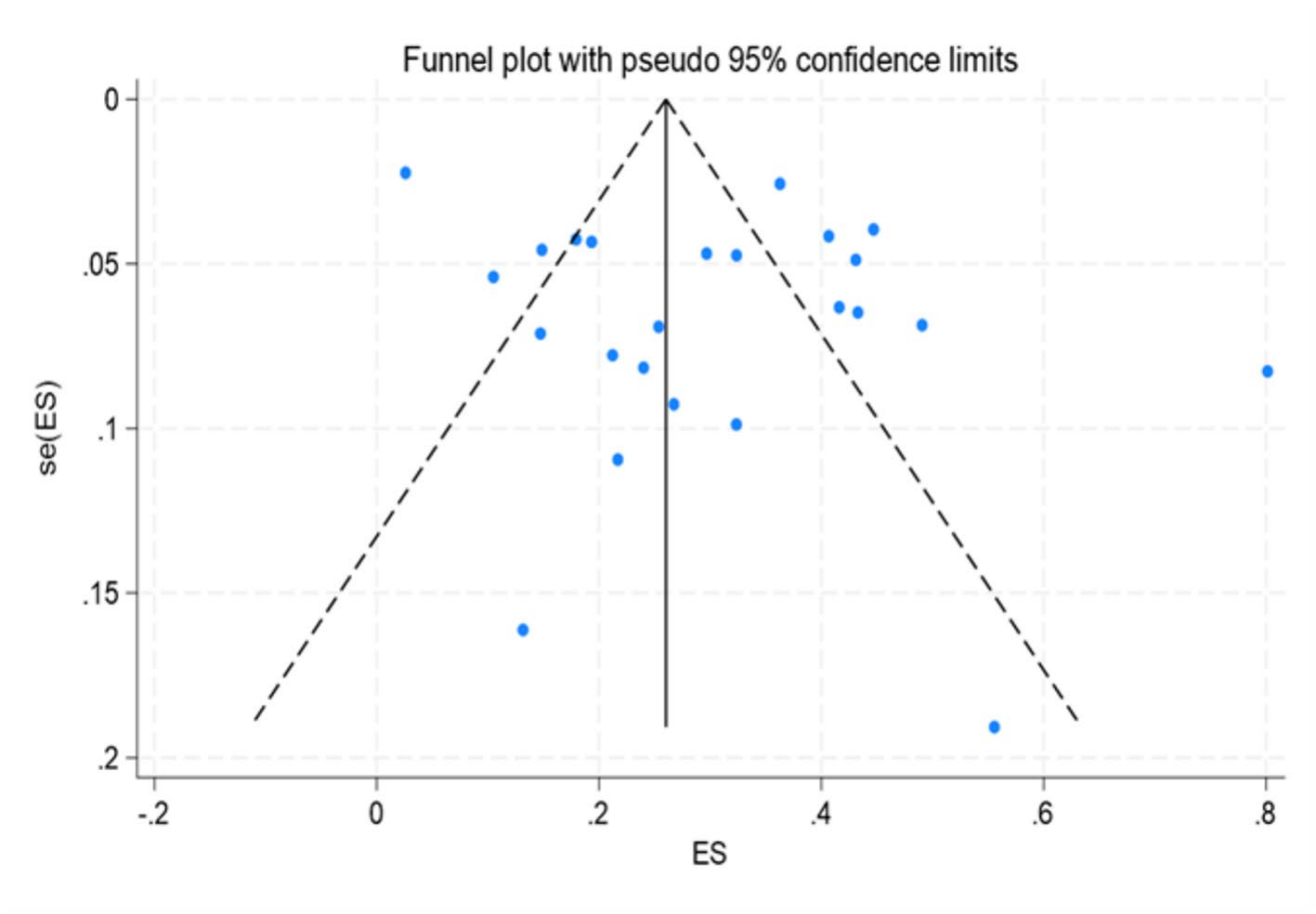
Meta-analysis funnel plot of incidence of oral mucosal pressure injury in patients undergoing endotracheal intubation.

The results of the meta-analysis of this study show that the incidence of OMPI in patients with oral tracheal intubation is 29%. However, the heterogeneity is relatively large. Further subgroup analysis results show that compared with traditional fixation methods, the incidence of OMPI in patients with oral tracheal intubation through decompression fixation is significantly lower. Decompression fixation mainly reduces the incidence of OMPI by using soft materials or preventive protection. The incidence of OMPI in patients with oral tracheal intubation in other countries is relatively higher than that in China. A cross-sectional study in China revealed that ICU nursing staff had a relatively low ability to recognize the early signs of OMPI, with accuracy rates regarding OMPI clinical staging and assessment tools being only 26 and 22%, respectively (35). This may cause some stage I OMPI to be overlooked, resulting in an OMPI incidence rate lower than the actual incidence rate. In addition, prospective cohort studies are more comprehensive and complete in terms of research design and data collection, and can detect OMPI more promptly. However, retrospective studies mainly rely on nursing records, which are prone to omissions, thus resulting in a relatively balanced incidence rate. ROMPIS is the first scale dedicated to oral mucosa, with more accurate diagnostic criteria and thus the closest to the true incidence rate. Due to the anatomical differences between the oral mucosa and the superficial skin, the NPIAP scale increases the incidence of OMPI, while the reliability of other scales remains to be verified and is prone to omissions. Nurses’ relatively frequent evaluations can detect more transient and early reversible mucosal ischemic changes, thereby increasing the incidence rate. Therefore, nursing staff can develop a structured assessment process, shorten the assessment intervals and appropriately increase the frequency of oral care based on the assessment results, achieving a closed-loop management of assessment –intervention – reassessment (Figure 3).

### 4.1 Intrinsic factors

The APACHE II score is an indicator for evaluating the severity of a patient’s condition. The higher the APACHE II score, the greater the risk of OMPI in patients. Patients with a high APACHE II score have severe and complex conditions, and there are common sensory disorders, insufficient perfusion, etc., thus increasing the risk of OMPI in patients (16, 36). Medical staff need to use the APACHE II scoring scale in a standardized manner to ensure the timeliness and accuracy of the assessment (37). For patients with high scores, strengthen multidisciplinary cooperation and actively adopt effective support methods to maintain the stability of patients’ vital signs and various systems.

People with diabetes are at high risk of developing OMPI. Vasculopathy associated with chronic hyperglycemia mainly result from the glycation of hemoglobin, the narrowing of blood vessels and the structural changes of red blood cell membranes (38). These vascular lesions not only exacerbate the damage to sensory nerves but also complicate the environment of the wound mucosa when bacterial infections occur inside the oral cavity. Due to persistent ischemia, hypoxia and the intensification of inflammatory responses, the normal healing process of wounds will be severely hindered. The nursing team should enhance their awareness of the full-process management of diabetic patients, monitor blood sugar dynamically, and effectively control blood sugar levels.

The research results show that for every 1 g/LOMPI increase in serum albumin, the risk decreases by 63%. Serum albumin is the main substance that maintains the colloid osmotic pressure of plasma. A decrease in its level will cause the colloid osmotic pressure of plasma to drop, leading to excessive water entering tissues and mucous membranes and causing edema (24). It is one of the nutritional indicators reflecting patients, and malnutrition can lead to a decline in the body’s immunity and a weakening of tissue repair capacity. It is one of the many independent nutritional risk factors that increase the risk of stress injury or interfere with the wound healing process (39). Stratified care is provided for patients with nutritional risks through nutritional screening tools. Interdisciplinary teams are collaborated with the nutrition department and others to develop early nutritional support plans for patients with oral tracheal intubation.

### 4.2 Treatment factors

This study shows that prone ventilation is one of the risk factors for OMPI in patients. During prone ventilation, due to changes in body position and center of gravity, the face may be compressed and the pressure distribution may be uneven. The oral mucosa is subjected to double compression by the intubation and its fixation device as well as the pressure from the head. Moreover, prone ventilation lasts for a relatively long time, averaging 8 to 24 h (39, 40). Studies have shown that applying pressure to mucosal tissue for 2 h can lead to irreversible ischemic damage to the tissue (41).

Vasoconstrictors are mainly used to increase mean arterial pressure and improve ischemia and hypoxia in vital organs of the body. They are common first-line drugs in clinical practice (2). Patients with oral tracheal intubation often need to use vasoactive drugs due to their condition to increase blood pressure and improve tissue perfusion. However, the use of vasoactive drugs can also cause the contraction of capillaries under the oral mucosa and aggravate insufficient tissue perfusion, thereby increasing the risk of OMPI. Mahmoodpoor et al. (42) demonstrated that patients receiving high-dose vasoconstrictor drugs had a nearly fourfold increased risk of pressure injury compared to those using low or moderate doses. For patients with oral tracheal intubation who use vasoconstrictor drugs, it is not only necessary to simply focus on the mean arterial pressure, but also to comprehensively assess the improvement of microcirculation and the condition of oral mucosa throughout the entire process, and to monitor and adjust the dosage in a timely manner to prevent excessive vasoconstriction leading to OMPI.

The use of anticoagulant drugs is a risk factor for OMPI. By directly acting on the key links of the coagulation waterfall, it reduces thrombosis, but it also increases the risk of bleeding (43). It promotes the aggregation of neutrophils on the walls of capillaries, generates a large number of oxygen free radicals locally in the mucosa, damages vascular endothelial cells, leads to microvascular dysfunction within the mucosa, reduces adaptability to external pressure, and makes it more prone to damage (44). For patients at risk of bleeding, it is necessary to conduct a comprehensive assessment of the bleeding risk, weigh the necessity of anticoagulant therapy, carefully select anticoagulant drugs, and at the same time strengthen health education and medication management for patients.

The research results show that patients using sedative drugs have a significantly increased risk of developing OMPI. During the process of continuous mechanical ventilation, patients usually need sedation to ensure the tolerance of oral tracheal intubation. However, sedative drugs can affect perception and autonomous response ability, and patients cannot express their discomfort in the oral cavity in a timely manner, which delays the attention, prevention and treatment of OMPI by nursing staff (45). Therefore, nursing staff should strive to minimize the inhibitory effect of sedative drug use on patients’ perception and response ability while ensuring their safety and comfort.

### 4.3 Device-related factors

Leaving a tracheal tube in place for too long can disrupt the normal physiological barrier function in the oral cavity, thereby increasing the risk of pressure injury. The longer the tracheal intubation is used, the more it compresses the local mucosa, resulting in tissue cell deformation, inflammatory edema, local ischemia, and hypoxia, eventually leading to OMPI (46). Cai et al. (29) conducted a study which demonstrated that a study shows that for every additional day of indwelling time of tracheal intubation, the risk of tracheal intubation-related pressure injury increased by 25.4%. Unlike skin, the oral mucosa has a low degree of keratinization and lacks the toughness and elasticity of skin, making it more vulnerable to damage from factors such as pressure and friction.

In addition, the use of hard dental pads increases the risk of OMPI in patients. On the one hand, when patients are intolerant to tracheal intubation and experience emotions such as anxiety, fear, and restlessness, it may cause them to swallow, bite, or resist the tracheal intubation and its fixation device, thereby increasing the pressure and friction of the dental pads on the oral mucosal tissue (47). On the other hand, dental pads and tracheal tubes are made of plastic or hard materials, which increases the risk of OMPI (4).

Meanwhile, the research found that the larger the model of tracheal intubation, the greater the risk of OMPI occurrence. The larger the intubation model, the greater the compression range of the oral mucosa. Coupled with the relatively weak local tissue, it is more likely to cause the occurrence of OMPI (48). Nursing staff should conduct a dynamic and comprehensive assessment of patients with oral tracheal intubation, select the appropriate tracheal intubation model and fix it reasonably. The tracheal intubation position should be re-fixed at least once per shift (2, 8). When conditions permit, silicone dental pads and decompression fixation methods can be considered to enhance patient comfort and the execution ability of nursing staff. At the same time, patients are encouraged to actively engage in weaning training to strive for early weaning and extubation, thereby reducing the risk of complications such as OMPI.

This study also has the following limitations: Most of the included studies are from China, which may lead to insufficient representativeness of the research results. The number of literature related to some risk factors is relatively small, which may have a certain impact on this research; The original studies included did not adequately report the characteristics of nursing staff, the reliability among evaluators, and the multidisciplinary collaboration model, which might be important sources of heterogeneity, but we were unable to further explore them through subgroup analysis. Future research should follow more comprehensive norms for nursing practice reporting.

## 5 Conclusion

The results of this study show that the incidence of oral mucosal pressure injury in patients with oral tracheal intubation is 29%. APACHEII score, diabetes, the use of anticoagulant drugs, the use of sedative drugs, the use of vasoconstrictor drugs, prone ventilation, the indwelling time of tracheal intubation, the use of hard dental pads, and the type of tracheal intubation are all positively correlated with the occurrence of OMPI, while blood white eggs are protective factors. Based on the above risk factors and in combination with the specific conditions of the patients, nursing staff can, through multidisciplinary collaboration, formulate a full-cycle, refined and personalized early intervention plan and optimize the oral care process for patients with tracheal intubation, in order to reduce the incidence of OMPI in patients with oral tracheal intubation.

## Data Availability

The original contributions presented in the study are included in the article/Supplementary material, further inquiries can be directed to the corresponding author.
