# Supplementary material for: Incidence and risk factors of oral mucosal pressure injury in patients with oral tracheal intubation: systematic review and meta-analysis
Source: Front Med (Lausanne). 2026 Apr 13;13:1783726. doi: 10.3389/fmed.2026.1783726 (PMC13111199; doi:10.3389/fmed.2026.1783726)
Supplement: Supplementary file 1 [file Data_Sheet_1.PDF]

Date searched: February 27, 2026

Database Searches and number of records identified:

1. PubMed:106
2. Embase:230
3. Web of Science:2627
4. Cochrane Library:261
5. Wanfang:176
6. VIP:25
7. CNKI:128
8. SinoMed:181

Detailed search strategy

1.PubMed

#1 ("Intubation, Intratracheal"[Mesh]) OR (Intratracheal Intubation[Title/Abstract] OR Intratracheal Intubations[Title/Abstract] OR Intubations, Intratracheal[Title/Abstract] OR Intubation, Endotracheal[Title/Abstract] OR Endotracheal Intubation[Title/Abstract] OR Endotracheal Intubations[Title/Abstract] OR Intubations,Endotracheal[Title/Abstract] OR Mechanical Ventilation[Title/Abstract] OR artificial airway[Title/Abstract])

#2 ("Pressure Ulcer"[Mesh]) OR (Pressure Ulcers[Title/Abstract] OR Ulcer, Pressure[Title/Abstract] OR medical device related pressure injuries[Title/Abstract] OR device-related ulcer[Title/Abstract] OR Decubitus Ulcer[Title/Abstract] OR Decubitus Ulcers[Title/Abstract] OR Ulcer,Decubitus[Title/Abstract] OR Pressure Sore[Title/Abstract] OR Pressure Sores[Title/Abstract] OR Sore, Pressure[Title/Abstract] OR Decubitus Sore[Title/Abstract] OR Decubitus Sores[Title/Abstract] OR Sore, Decubitus[Title/Abstract] OR Pressure Injury[Title/Abstract] OR Injury, Pressure[Title/Abstract] OR Pressure Injuries[Title/Abstract] OR oral mucosal pressure injury[Title/Abstract] OR mucosal pressure injury[Title/Abstract])

#3 ("Risk Factors"[Mesh]) OR (risk factor[Title/Abstract] OR factor*,social risk[Title/Abstract] OR risk factor*,social[Title/Abstract] OR health correlates[Title/Abstract] OR population*at risk[Title/Abstract] OR risk score*[Title/Abstract] OR risk factor score*[Title/Abstract] OR influence*factor*[Title/Abstract] OR relevant factor*[Title/Abstract] OR predict* factor*[Title/Abstract])

#4 #1 AND #2 AND #3

2.Embase

#1 'endotracheal intubation'/exp OR 'intratracheal intubation':ab,kw,ti OR ‘Intratracheal Intubation’:ab,kw,ti OR ‘Intratracheal Intubations’:ab,kw,ti OR ‘Intubations, Intratracheal’:ab,kw,ti OR ‘Intubation, Endotracheal’:ab,kw,ti OR ‘Endotracheal Intubation’:ab,kw,ti OR ‘Endotracheal Intubations’:ab,kw,ti OR ‘Intubations, Endotrachea’:ab,kw,ti OR ‘Mechanical Ventilation’:ab,kw,ti OR ‘Artificial airway’:ab,kw,ti

#2 'decubitus'/exp OR ‘Pressure Ulcers':ab,kw,ti OR ‘Ulcer, Pressure':ab,kw,ti OR ‘medical device related pressure injuries ':ab,kw,ti OR ‘device-related ulcer ':ab,kw,ti OR ‘Decubitus Ulcer':ab,kw,ti OR ‘Decubitus Ulcers':ab,kw,ti OR ‘Ulcer, Decubitus':ab,kw,ti OR ‘Pressure

Sore':ab,kw,ti OR ‘Pressure Sores':ab,kw,ti OR ‘Sore, Pressure':ab,kw,ti OR ‘Decubitus Sore':ab,kw,ti OR ‘Decubitus Sores':ab,kw,ti OR ‘Sore, Decubitus':ab,kw,ti OR ‘Pressure Injury':ab,kw,ti OR ‘Injury, Pressure':ab,kw,ti OR ‘Pressure Injuries':ab,kw,ti OR ‘oral mucosal pressure injury':ab,kw,ti OR ‘mucosal pressure injury':ab,kw,ti

#3 'risk factor'/exp OR ‘risk factors’:ab,kw,ti OR ‘factor*,social risk’:ab,kw,ti OR ‘risk factor*,social’:ab,kw,ti OR ‘health correlates’:ab,kw,ti OR ‘population*at risk’:ab,kw,ti OR ‘risk score*’:ab,kw,ti OR ‘risk factor score*’:ab,kw,ti OR ‘influence*factor*’:ab,kw,ti OR ‘relevant factor*’:ab,kw,ti OR ‘predict* factor*’:ab,kw,ti

#4 #1 AND #2 AND #3

3. Web of Science

TS=(Intubation, Intratracheal OR Intratracheal Intubation OR Intratracheal Intubations OR Intubations, Intratracheal OR Intubation, Endotracheal OR Endotracheal Intubation OR Endotracheal Intubations OR Intubations, Endotracheal OR Mechanical Ventilation OR Artificial airway) AND TS=(Pressure Ulcer OR Pressure Ulcers OR Ulcer, Pressure OR medical device related pressure injuries OR device-related ulcer OR Decubitus Ulcer OR Decubitus Ulcers OR Ulcer, Decubitus OR Pressure Sore OR Pressure Sores OR Sore, Pressure OR Decubitus Sore OR Decubitus Sores OR Sore, Decubitus OR Pressure Injury OR Injury, Pressure OR Pressure Injuries OR oral mucosal pressure injury OR mucosal pressure injury) AND TS=(Risk Factors OR risk factor OR factor*,social risk OR risk factor*,social OR health correlates OR population*at risk OR risk score* OR risk factor score* OR influence*factor* OR relevant factor* OR predict* factor*)

4. Cochrane Library

#1 MeSH descriptor: [Intubation, Intratracheal] explode all trees

#2 (intratracheal intubation):ab,kw,ti OR (Intratracheal Intubation):ab,kw,ti OR (Intratracheal Intubations):ab,kw,ti OR (Intubations, Intratracheal):ab,kw,ti OR (Intubation, Endotracheal):ab,kw,ti OR (Endotracheal Intubation):ab,kw,ti OR (Endotracheal Intubations):ab,kw,ti OR (Intubations, Endotracheal):ab,kw,ti OR (Mechanical Ventilation):ab,kw,ti OR (Artificial airway):ab,kw,ti

#3 #1 OR #2

#4 MeSH descriptor: [Pressure Ulcer] explode all trees

#5 (Pressure Ulcers):ab,kw,ti OR (Ulcer, Pressure):ab,kw,ti OR (medical device related pressure injuries):ab,kw,ti OR (device-related ulcer):ab,kw,ti OR (Decubitus Ulcer):ab,kw,ti OR (Decubitus Ulcers):ab,kw,ti OR (Ulcer, Decubitus):ab,kw,ti OR (Pressure Sore):ab,kw,ti OR (Pressure Sores):ab,kw,ti OR (Sore, Pressure):ab,kw,ti OR (Decubitus Sore):ab,kw,ti OR (Decubitus Sores):ab,kw,ti OR (Sore, Decubitus):ab,kw,ti OR (Pressure Injury):ab,kw,ti OR (Injury, Pressure):ab,kw,ti OR (Pressure Injuries):ab,kw,ti OR (oral mucosal pressure injury):ab,kw,ti OR (mucosal pressure injury):ab,kw,ti

#6 #4 OR #5

#7 MeSH descriptor: [Risk Factors] explode all trees

#8 (risk factor):ab,kw,ti OR (factor*,social risk):ab,kw,ti OR (risk factor*,social):ab,kw,ti OR (health correlates):ab,kw,ti OR (population*at risk):ab,kw,ti OR (risk score*):ab,kw,ti OR (risk

factor score*):ab,kw,ti OR (influence*factor*):ab,kw,ti OR (relevant factor*):ab,kw,ti OR (predict* factor*):ab,kw,ti
#7 #3 AND #6 AND #9

5.Wanfang
主题=(气管插管 OR 经口气管插管 OR 机械通气 OR 人工气道) AND 主题=(口腔黏膜压力性损伤 OR 黏膜压力性损 OR 压力性损伤 OR 压疮 OR 压力性溃疡 OR 医疗器械相关压力性损伤 OR 器械性压力损伤 OR 受压溃疡) AND 主题=(危险因素 OR 影响因素 OR 相关因素 OR 预测因素)

6.VIP
题名或关键词=(气管插管 + 经口气管插管 + 机械通气 + 人工气道) AND 题名或关键词=(口腔黏膜压力性损伤 +黏膜压力性损伤 + 压力性损伤 + 压疮 + 压力性溃疡 + 医疗器械相关压力性损伤 + 器械性压力损伤 + 受压溃疡) AND 题名或关键词=(危险因素 + 影响因素 + 相关因素 + 预测因素)

7. CNKI
主题=(气管插管 + 经口气管插管 + 机械通气 + 人工气道） AND 主题=(口腔黏膜压力性损伤 + 黏膜压力性损伤 + 压力性损伤 + 压疮 + 压力性溃疡 + 医疗器械相关压力性损伤 + 器械性压力损伤 + 受压溃疡) AND 主题=(危险因素 + 影响因素 + 相关因素 + 预测因素)

8. SinoMed
常用字段=（气管插管 OR 经口气管插管 OR 机械通气 OR 人工气道） AND 常用字段=（口腔黏膜压力性损伤 OR 黏膜压力性损 OR 压力性损伤 OR 压疮 OR 压力性溃疡 OR 医疗器械相关压力性损伤 OR 器械性压力损伤 OR 受压溃疡) AND 常用字段=(危险因素 OR 影响因素 OR 相关因素 OR 预测因素)
